# Carbon Ion Radiotherapy Induce Metabolic Inhibition After Functional Imaging-Guided Simultaneous Integrated Boost for Prostate Cancer

**DOI:** 10.3389/fonc.2022.845583

**Published:** 2022-07-22

**Authors:** Yulei Pei, Renli Ning, Wei Hu, Ping Li, Zhenshan Zhang, Yong Deng, Zhengshan Hong, Yun Sun, Xiaomao Guo, Qing Zhang

**Affiliations:** ^1^ Department of Radiation Oncology, Shanghai Proton and Heavy Ion Center, Fudan University Cancer Hospital, Shanghai, China; ^2^ Shanghai Key Laboratory of Radiation Oncology (20dz2261000), Shanghai, China; ^3^ Shanghai Engineering Research Center of Proton and Heavy lon Radiation Therapy, Shanghai, China; ^4^ Department of Research and Development, Shanghai Proton and Heavy Ion Center, Fudan University Cancer Hospital, Shanghai, China; ^5^ Department of Radiation Oncology, Shanghai Proton and Heavy Ion Center, Shanghai, China; ^6^ Department of Research and Development, Shanghai Proton and Heavy Ion Center, Shanghai, China

**Keywords:** simultaneous integrated boost, carbon ion radiotherapy, metabolite profiles, prostate cancer, local effect model

## Abstract

**Purpose:**

As local recurrence remains a challenge and the advantages of the simultaneous integrated boost (SIB) technique have been validated in photon radiotherapy, we applied the SIB technique to CIRT. The aim was to investigate the metabolomic changes of the CIRT with concurrent androgen deprivation therapy (ADT) in localized prostate cancer (PCa) and the unique metabolic effect of the SIB technique.

**Material and Methods:**

This study enrolled 24 pathologically confirmed PCa patients. All patients went through CIRT with concurrent ADT. The gross target volume (GTV) boost was defined as positive lesions on both ^68^Ga-PSMA PET/CT and mpMRI images. Urine samples collected before and after CIRT were analyzed by the Q-TOF UPLC-MS/MS system. R platform and MetDNA were used for peak detection and identification. Statistical analysis and metabolic pathway analysis were performed on Metaboanalyst.

**Results:**

The metabolite profiles were significantly altered after CIRT. The most significantly altered metabolic pathway is PSMA participated alanine, aspartate and glutamate metabolism. Metabolites in this pathway showed a trend to be better suppressed in the SIB group. A total of 11 identified metabolites were significantly discriminative between two groups and all of them were better down-regulated in the SIB group. Meanwhile, among these metabolites, three metabolites in DNA damage and repair related purine metabolism were down-regulated to a greater extent in the SIB group.

**Conclusion:**

Metabolic dysfunction was one of the typical characteristics of PCa. CIRT with ADT showed a powerful inhibition of PCa metabolism, especially in PSMA participated metabolic pathway. The SIB CIRT showed even better performance on down-regulation of most metabolism than uniform-dose-distribution CIRT. Meanwhile, the SIB CIRT also showed its unique superiority to inhibit purine metabolism. PSMA PET/CT guided SIB CIRT showed its potentials to further benefit PCa patients.

## Introduction

Prostate cancer (PCa) ranked as the fourth common malignancy worldwide (1) poses major social and family problems. Carbon ion radiotherapy (CIRT) is an emerging cancer treatment strategy (2–6) for PCa patients which has a stronger effect on tumor cells per physical dose (higher relative biological effect) and better dose distribution compared with photon-based radiotherapies (7). Numerous studies (8–11) from Japan reported that CIRT may improve 5- and 10-year progression-free survival rates with favorable late genitourinary and gastrointestinal toxicities compared with photon radiotherapy.

For definitive localized PCa irradiation, the clinical target volume (CTV) is mainly defined as the entire prostate and part of the seminal vesicle with a uniform dose delivery. Nonetheless, the local recurrence remains a challenge and is still under investigation, for several studies (12, 13) have shown that local recurrences after definitive radiotherapy mainly occur in the primary imaging detectable site, which is considered as the high-risk area with local recurrence or residual disease. A higher irradiation dose to the area with the highest risk for tumor persistence may increase local control (14). Every 1 Gy dose escalation of the total irradiation dose may benefit the improvement of 1.8% biochemical-free survival (15). To further improve the dose distribution on the tumor, the simultaneous integrated boost (SIB) technique was developed and employed to deliver a higher radiation dose to the specific ‘at risk’ area with an acceptable organ at risk (OAR) dose (16). Previously published data (17) reported that the PSMA-PET/CT-guided SIB in photon radiotherapy has favorable infield control for men with oligorecurrent PCa. The 3-year biochemical progression-free survival and overall survival (OS) were 55% and 84%, respectively. Until now, there has been no report about the application of SIB in CIRT.

To our knowledge, PCa has typical and unique metabolic profiles, with dysregulation in polyamines, tricarboxylic acid (TCA) cycle, amino acids, and fatty acids metabolism (18), and some of the dysregulated metabolism are highly related to carcinogenesis and progression (19, 20). Inhibiting some of the abnormally up-regulated pathways could bring significant metabolic effects and therapeutic potential (21). Some studies have evaluated the treatment-induced metabolic alternation and tumor metabolism is now developing into an assessment tool for therapeutic treatment efficacy (22, 23). Pickett et al. (23) used endorectal magnetic resonance spectroscopy imaging to compare the three-dimensional conformal external beam radiotherapy with permanent prostate implantation (PPI) and found that PPI could be more effective at destroying prostate metabolism along with better biochemical responses. Thus, tumor metabolism could be a feasible way to assess the real-time treatment response between the different treatment strategies.

As the most downstream of omics cascade, metabolomics provides a comprehensive description of individual phenotypes. Previous studies have investigated the metabolomic changes after several therapeutic strategies: neoadjuvant chemotherapy with androgen deprivation therapy (ADT) (24), radical prostatectomy (25) as well as photon radiation (26, 27), and the results pointed out that the metabolite profiles had altered to varying levels after receiving the treatment. However, metabolomic changes after radical radiation therapy with ADT have not been investigated yet, especially in CIRT, as well as the SIB strategy. The irradiation responses were also highly correlated with metabolism (28), and targeting tumor metabolism could overcome the radio-resistance (29, 30). A previous study (31) showed that carbon-ion irradiation could down-regulate several metabolites levels in the tumor, while photon radiation would up-regulate them. Also, our last study pointed out that CIRT generated metabolic reprogramming and induced individualized response in PCa. Thus, we built an ultrasensitive metabolites detection platform based on UPLC-MS/MS to explore the metabolic alteration by two CIRT strategies with concurrent ADT in PCa patients.

## Materials and Methods

### Patients Characteristics and Staging Evaluation

**Figure 1:**
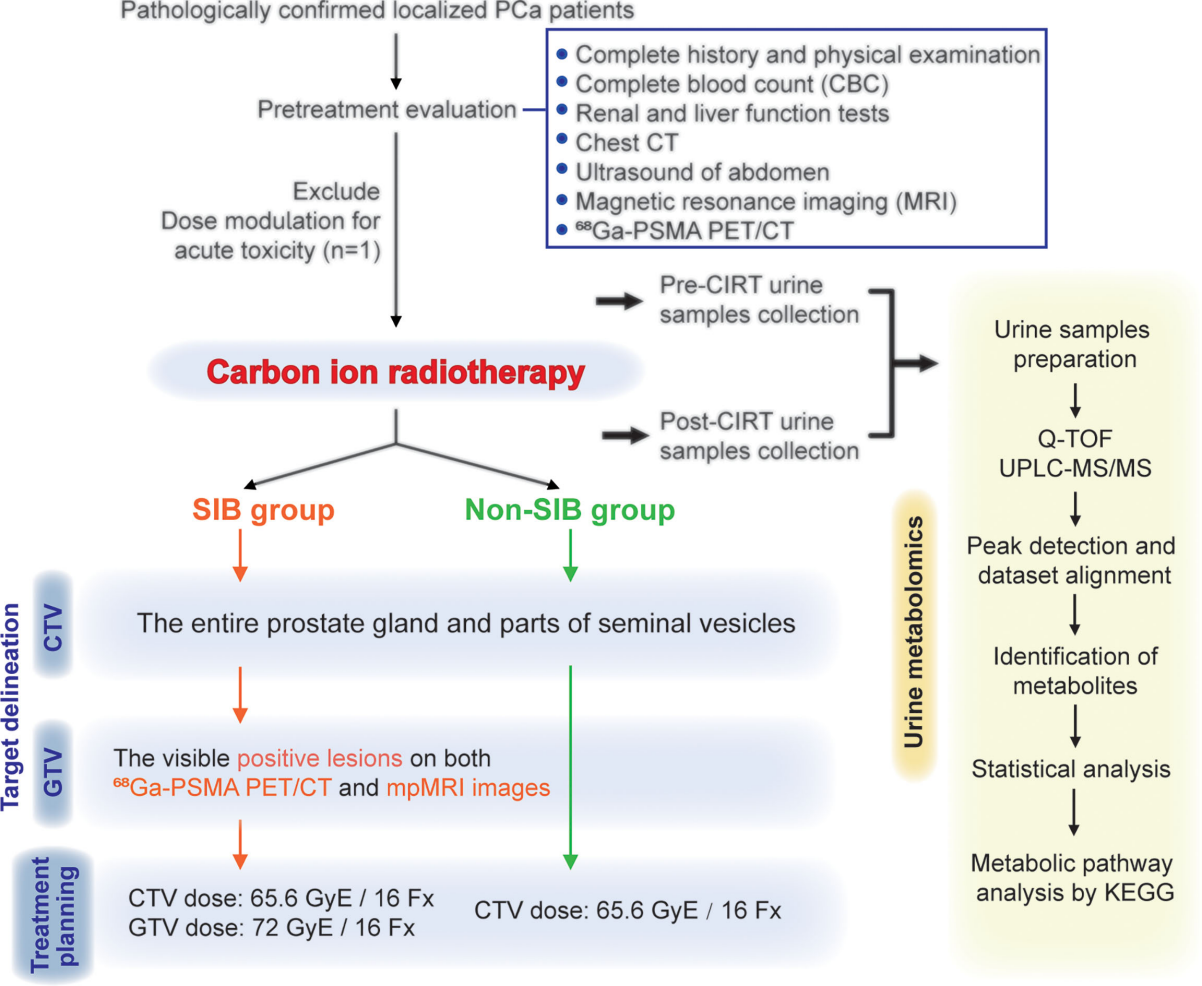
Flowchart of patients. This study enrolled 24 pathologically confirmed localized PCa patients. The delineation of CTVs were consistent in the two groups. Patients in the SIB group received the SIB on positive lesions that were visible on both ^68^Ga-PSMA PET/CT and mpMRI images. Urine samples were collected at the start and the end of CIRT, and then went through the metabolomic analysis process.

From July 2020 to May 2021, 24 patients pathologically confirmed of staged II to III (American Joint Committee on Cancer TNM Staging System and Prognostic Groups for prostate cancer, 8th edition, 2017) prostate adenocarcinoma were enrolled in this study. One patient dropped due to acute toxicity, and finally, 23 patients were included in this analysis. The flow chart of this study is shown in **Figure 1**. Pretreatment evaluation consisted of a complete history and physical examination, complete blood count (CBC), renal and liver function tests, chest CT, ultrasound of the abdomen, magnetic resonance imaging (MRI), and ^68^Ga-PSMA PET/CT. Patient characteristics are summarized in **Table 1**.

**Table 1:** Patients’ demographic and clinical characteristics.

Characteristics	Statistics	All patients	Non-SIB group	SIB group	p value^#^
		No. of patients (n = 24)	No. of patients (n = 13)	No. of patients (n = 7)	
Age (years)		75.5 (56-84)	78 (56-84)	73.5(71-81)	0.525
T					0.690
	T2	16 (66.67%)	8 (61.54%)	4 (57.14%)	
	T3	7 (29.17%)	4 (30.77%)	3 (42.86%)	
	Tx	1 (4.17%)	1 (7.69%)	0 (0.00%)	
N	N0	24 (100%)	13(100.00%)	7(100.00%)	NA
M	M0	24 (100%)	13(100.00%)	7(100.00%)	NA
Gleason Score					0.493
	6	5 (20.83%)	4 (30.77%)	1 (14.29%)	
	7	11 (45.83%)	4 (30.77%)	4 (57.14%)	
	≥8	8 (33.33%)	5 (38.46%)	2 (28.57%)	
Risk Group					0.690
	Low	0 (0.00%)	0 (0.00)	0 (0.00%)	
	Intermediate	10 (41.67%)	4 (30.77%)	3 (42.86%)	
	High	13 (54.17%)	8 (61.54%)	4 (57.14%)	
	Very high	1 (4.17%)	1 (7.69%)	0 (0.00%)	
ADT	Neoadjuvant ADT^*^	24 (100%)	13 (100%)	7 (100%)	NA

T, tumor; N, lymph node; M, metastasis; ADT, androgen deprivation therapy; NA, not applicable.

*Patients underwent neoadjuvant ADT 2-3 months before CIRT, and there was no significant difference (p=0.4251) between the two groups in the duration of ADT before CIRT.

^#^The p-value demonstrated the difference of characteristics between the non-SIB group and the SIB group.

### Carbon Ion Radiotherapy Treatment

#### Target Delineation

The treatment included two phases. CTV1 was defined as the entire prostate gland and inferior 1-1.5cm of seminal vesicles for intermediate-risk patients, and the entire prostate gland and inferior 2-2.5cm of seminal vesicles for high- and very high-risk patients. The shrink-down strategy was then used to spare bladder and rectum, while CTV2 was defined as the entire prostate gland. The delineation of the CTVs was consistent in both groups. The delineation of the CTVs complied with the ESTRO ACROP consensus guideline and was consistent in both groups (32). The GTV was defined as the original positive lesion that is visible on both ^68^Ga-PSMA PET/CT and mpMRI images.

**Figure 2:**
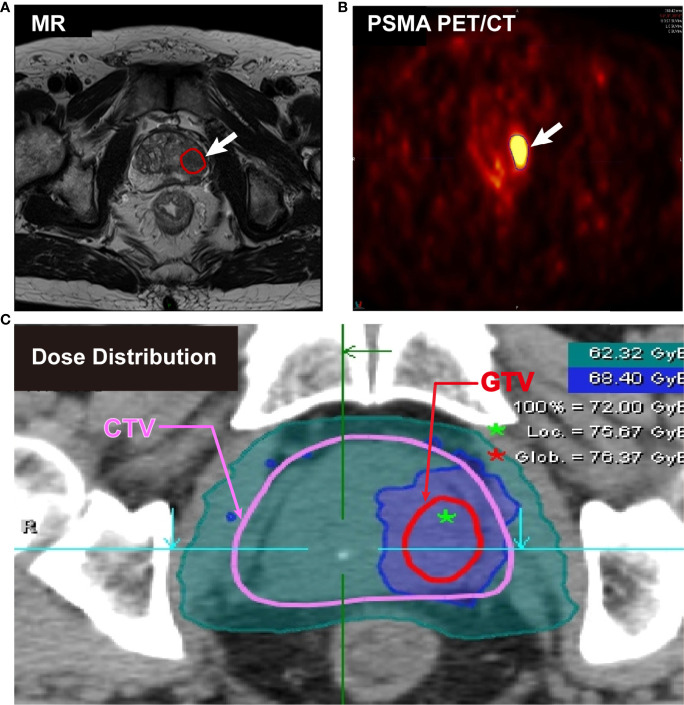
Imaging examination and delineation of tumor volume from a cT3aN0M0 patient. **(A)** Positive PCa lesions on MR image. **(B)** High uptake positive lesions on ^68^Ga-PSMA PET/CT image. **(C)** Clinical target volume (CTV) (red) and gross target volume (GTV) (pink) for simultaneous integrated boost (SIB) on planning CT.

The determination of the gross target volume (GTV) boost was critical work in CIRT. Normally, multiparametric magnetic resonance imaging (mpMRI) is recommended by the European Association of Urology and the European Society of Urogenital Radiology (EAU-EANM-ESTRO-ESUR-SIOG Guidelines on Prostate Cancer, 2020), the National Comprehensive Cancer Network (NCCN Clinical Practice Guideline in Oncology, 2020) for PCa diagnosis. mpMRI is prominent in high soft-tissue contrast and is not only able to portray detailed distinction between the prostate and periprostatic structures but also clearly visualize intraprostatic lesions, which is proved to benefit the accuracy in radiotherapy planning. PSMA PET/CT is another advanced technique for PCa detection. Over 90% of intraprostatic tumor lesions are positive in ^68^Ga-PSMA PET/CT imaging (33). ^68^Ga-PSMA-PET defined GTV was twice the volume (median 4.9 mL vs 2.8 mL) than mpMRI (34). Using PSMA PET/CT for target delineation in dose painting has been proved to benefit the tumor control probability without increasing normal tissue complication probability (NTCP). In combination with the advantages of mpMRI and PSMA PET/CT, Zamboglou et al. (35) applied these two techniques into the planning system and found it feasible to use the union of both PSMA-PET/CT and mpMRI based GTV for a focal boost with or without a minimal increase of NTCP compared to the GTV based on only PSMA PET/CT or only mpMRI. In our study, the GTV was contoured by at least two associate professors. The imaging examination and delineation of tumor volume are presented in **Figure 2**.

#### Treatment Planning

The CIRT was delivered by the Siemens IONTRIS particle therapy system. Syngo (V13B, Siemens, Germany) with the local effect model (LEM) I was used for the treatment planning. The patients received the following irradiation doses: 55.2 GyE in 12 fractions (4.6 GyE per daily fraction), 65.6-72 GyE in 16 fractions (4.1-4.5 GyE per daily fraction). Patients with 55.2 GyE irradiation were included in the overall analysis and excluded in the comparison of the SIB group and the uniform-dose-distribution group (non-SIB group).

For the SIB group and the non-SIB group, the prescription dose of the entire prostate gland was 65.6 GyE in 16 fractions and delivered within two phases. The total dose of 49.2GyE in 12 fractions was prescribed to CTV1 in the first phase and the prescription dose of 16.4GyE in 4 fractions was delivered to CTV2 in the second phase. For the SIB group, a total dose of 72 GyE in 16 fractions to GTV was simultaneously boosted, and the prescription dose to GTV was consistent. 95% of the prescribed dose should cover 100% of the CTVs and 90% of the isodose line should cover 100% of the PTVs. Bladder and rectum preparation were performed before each irradiation, and both in-room CT and 2D PORT film were performed for position matching. The standard for position error should be meet the following criteria: X, Y, and Z directions error should be less than 0.3cm, and the rotation error should be less than 1° as well. A 3mm margin outside GTV was also set up to make sure that the positive lesion could be fully covered with a 95% isodose line in case there was any position error. The total ADT duration was based on different risk groups. Patients underwent neoadjuvant ADT 2-3 months before CIRT, followed by concurrent ADT with CIRT, and adjuvant ADT thereafter. There was no significant difference (p=0.4251) between the two groups in the duration of ADT before CIRT.

### Metabolomics Methods

#### Samples Collection and Processing

Urine samples were gathered within the 4 hours before receiving the first fraction and 4 hours after finishing the last fraction and then stored in a 4°C fridge immediately. Contaminated bacteria were removed by 0.22 μm membrane filters. 800 μL chilled methanol/acetonitrile (1:1, v/v) mixed solution was added to each 200 μL thawed sample. The mixture was centrifuged and the supernatant was transferred into a new microcentrifuge tube, and then evaporated to dryness. Re-dissolved the dry substance into a 100 μL chilled mixture of acetonitrile/water (1:1, v/v), and then transferred the solution into sample vials. Quality control (QC) samples consisted of the mixture of equal amounts (50 μL) of each sample.

#### Q-TOF UPLC -MS/MS Analysis

The urine samples were analyzed by the high-throughput ultrasensitive liquid chromatography, linked to tandem mass spectrometry (AB SCIEX ExionLCY system combined with 500R QTOF). The ACQUITY UPLC BEH Amide 1.7 μm (2.1 mm × 100 mm) column was used to separate the urinary metabolites. Mobile phase A: a mixture of 20 mmol ammonium hydroxide and 20 mmol ammonium formate to 1 L water and mixing thoroughly. Mobile phase B: acetonitrile. The flow program was set as follows: 0 min, 95% B; 14 min, 65% B; 16 min, 40% B; 18.1 min, 95% B; 23 min, 95% B. Electrospray ionization mode was used in the analysis. Auto-calibration was performed every five analysis. Four blanks and six QC samples were injected at the start for column conditioning, and the QC samples were analyzed every ten injections. Auto calibration was performed every five analyses.

#### Data Collection and Metabolites Identification

The raw data was got from a Q-TOF UPLC -MS/MS, and peak detection and dataset alignment were performed by the R platform. MetDNA was used to identify the metabolites. The generated peak tables were uploaded onto the Metaboanalyst (https://www.metaboanalyst.ca/MetaboAnalyst/ModuleView.xhtml) for statistical and metabolic pathway analysis. Concentrations of all metabolites were expressed as peak area and normalized according to the creatinine levels.

#### Statistical Analysis

The analysis of data was performed on MetaboAnalyst. To compare the difference of pre- and post-CIRT samples, the following statistical methods were applied. A volcano plot consisting of fold change (FC) analysis and Wilcoxon signed-rank test was used to identify statistically significantly different metabolites (FDR<0.05, FC>2) and the heat maps were performed to show the differences of metabolite profiles. Wilcoxon signed-rank test was used for statistical analysis, and the significance level was defined as p<0.05. The significant separation shift between compared groups was tested using the unsupervised principal component analysis (PCA). Supervised multivariate analysis by partial least-squares discriminant analysis (PLS-DA) was performed to achieve maximum separation among the groups. The pathway analysis was performed between pre-CIRT samples and post-CIRT samples, and the significance level was defined as FDR<0.05, Impact>0.2. Box plots showed the mean value with the standard deviation. To compare the difference between the non-SIB group and the SIB group, the following statistical methods were applied. The volcano plot was used to identify statistically significantly different metabolites (p<0.05, FC>2). Mann Whitney test and Pearson χ^2^ test were used for statistical analysis, and the significance level was defined as p<0.05.

## Results

### Inhibition Effect of CIRT on Localized PCa Metabolism

Urine samples from 23 patients were enrolled in the analysis. Among all patients, 13 patients received a 65.6 GyE uniform-dose-distribution CIRT and 7 patients received 65.6 GyE CIRT with a 72 GyE SIB to ^68^Ga-PSMA PET/CT and mpMRI visibly positive lesion. We tried to compare the metabolic effect of these two CIRT strategies. All patients’ urine samples were collected at the beginning and the end of the whole course and then monitored by high-throughput Q-TOF UPLC-MS/MS. Finally, a total of 6468 peaks were monitored in patients’ urine samples.

**Figure 3:**
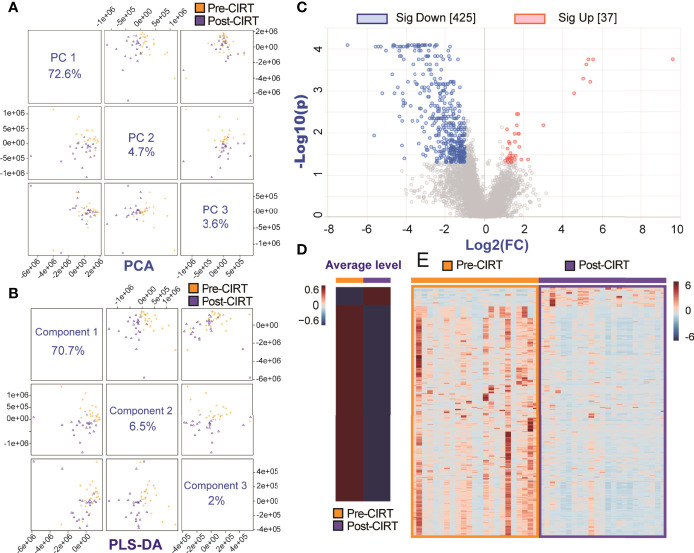
The metabolic inhibition effect of CIRT on PCa. The PCA **(A)** and PLS-DA **(B)** analysis of all pre-CIRT and post-CIRT samples from the both SIB group and non-SIB group. **(C)** The volcano plot of pre-CIRT and post-CIRT samples from all PCa patients in both groups. Blue and red dots represent significantly down-regulated and up-regulated metabolites, respectively (FDR<0.05, FC>2). **(D)** The heat map showed the average level of significantly altered metabolites in pre-CIRT and post-CIRT samples. **(E)** The heat map showed the detailed level of significantly altered metabolites in each pre-CIRT and post-CIRT sample.

To explore CIRT induced metabolic alternation, we took pre-CIRT and post-CIRT urine samples from all patients into metabolomic analysis. The PCA and the PLS-DA analysis showed a clear separation between pre-CIRT and post-CIRT samples, which meant that the metabolite profiles were significantly altered after CIRT (**Figures 3A, B**). **Supplementary Figure 1** and **Supplementary Table 1** showed PLS-DA cross validation details. All peaks were taken into volcano plot analysis to detect the significantly altered metabolites triggered by CIRT. Volcano plot identified 462 significantly altered metabolites, including 425 down-regulated ones and 37 up-regulated ones, which means 92.0% of the total significantly altered metabolites were down-regulated, indicating CIRT could significantly suppress or slow down most metabolism progressions (**Figure 3C**). The significantly altered identified metabolites were listed in **Supplementary Figure 2** and **Supplementary Table 2**. The heat map visually showed the variation of the significantly altered metabolites in all samples in both groups. **Figures 3D, E** respectively depicted the average level of pre-CIRT and post-CIRT samples and the detailed level of each sample collected before and after CIRT, respectively. Clearly, the production level of most significantly altered metabolites was down-regulated after CIRT.

### PCa Associated PSMA Participated Metabolism Pathway Tends to Be Suppressed to a Greater Extent in the SIB Group Than in the Non-SIB Group

**Figure 4:**
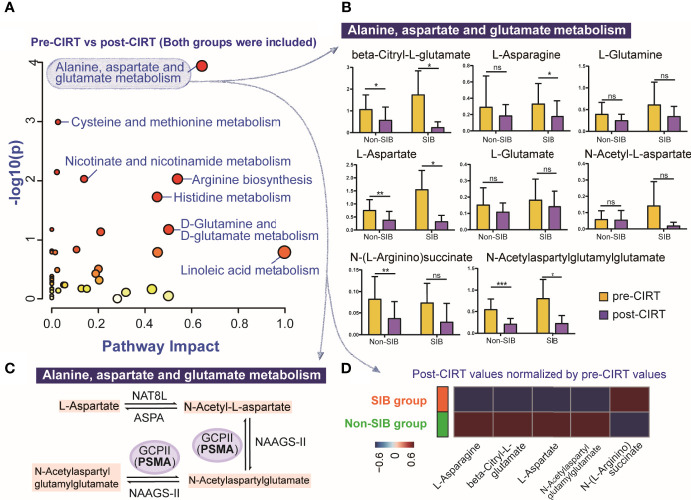
The compare of the most significantly altered pathway after between the SIB group and the non-SIB group. **(A)** Pathway analysis of all pre-CIRT and post-CIRT samples in both SIB group and non-SIB group. **(B)** Box plots of 8 identified metabolites in alanine, aspartate and glutamate metabolism pathway of pre-CIRT samples and post-CIRT samples in both groups. **(C)** Schematic diagram of the PCa associated PSMA participated metabolic pathway named alanine, aspartate and glutamate metabolism. **(D)** The heat map illustrated the comparation of the average level of post-CIRT values normalized by pre-CIRT values of significantly altered metabolites in alanine, aspartate and glutamate metabolism pathway between two groups.*p < 0.05; **p < 0.01; ***p < 0.001; ns, p > 0.05, no statistical difference.

The pathway analysis of urine metabolites between all pre-CIRT and post-CIRT samples indicated that alanine, aspartate and glutamate metabolism was the most significantly altered pathway (FDR= 0.0044804, impact= 0.64263), shown in **Figure 4A**. A total of 8 identified metabolites were enriched into this pathway. We compared alternation of these metabolites between the SIB group and the non-SIB group (**Figure 4B**). Four of eight metabolites were significantly down-regulated in the non-SIB group: L-aspartate, N-(L-arginino)succinate, beta-Citryl-L-glutamate, and N-acetylaspartylglutamylglutamate. Meanwhile, four of eight metabolites were down-regulated in the SIB group as well: L-aspartate, L-asparagine, beta-Citryl-L-glutamate, and N-acetylaspartylglutamylglutamate. As we further dug deeper into this pathway, we found that PSMA played a key role in regulating it. It works as a N-acetyl-L-aspartyl-L-glutamate peptidase that cleaves the terminal poly glutamates connected with N-Acetyl-L-aspartate. **Figure 4C** demonstrated the role of the PSMA (glutamate carboxypeptidase II, GCPII) in the alanine, aspartate and glutamate metabolism pathway. Post-CIRT values of significantly altered metabolites in the alanine, aspartate and glutamate metabolism pathway were normalized by the division of corresponding pre-CIRT values. The average level of normalized post-CIRT was used in the heat map to visually illustrate the difference of significantly altered metabolites in this pathway between the SIB group and the non-SIB group (**Figure 4D**). The majority (4/5) of the metabolites in this pathway showed a trend to be down-regulated to a lower level in the SIB group compared with the non-SIB group. This brought the fact that even a minor boost of 6.4 (0.4 GyE per fraction) GyE to PSMA positive PCa lesion could induce a powerful inhibition of PSMA participated metabolic pathway in PCa, indicating the SIB CIRT technique was more effective to localized PCa than uniform-dose-distribution CIRT.

### Better Inhibition Effects of the SIB CIRT Technique on Localized PCa Metabolism Compared to Uniform-Dose-Distribution CIRT

**Figure 5:**
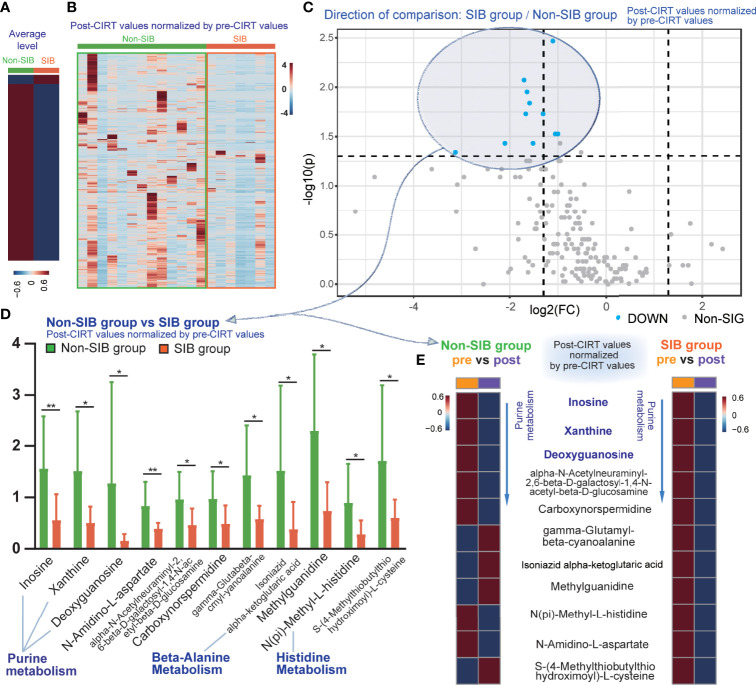
The inhibition effect of the SIB CIRT technique on PCa metabolism compared to uniform-dose-distribution CIRT. The heat map compared the average **(A)** and detailed **(B)** level of post-CIRT values normalized by pre-CIRT values between the two groups. **(C)** The volcano plot of the normalized post-CIRT values of the SIB group and the non-SIB group. Blue dots represented metabolites that had significantly lower concentrations in the SIB group compared with the non-SIB group. (p<0.05, FC>2) **(D)** Box plots of 11 identified significantly discriminative metabolites between the SIB group and the non-SIB group. **(E)** The heat map showed the average level of pre-CIRT samples and post-CIRT to compare the variation of these significantly discriminative metabolites in both groups, respectively.

Post-CIRT values were normalized by the division of the corresponding pre-CIRT values to compare the differences of metabolic effect between these two strategies. The heatmap of normalized post-CIRT values showed that the SIB group had overall lower metabolites level than the non-SIB group, indicating the SIB CIRT technique had better inhibition on most metabolism than uniform-dose-distribution CIRT. (**Figures 5A, B**). Then we compared normalized post-CIRT values between two groups. A total of 11 identified metabolites were significantly discriminative (p<0.05, FC>2) between the two groups, and all of them showed a lower level in the SIB group (**Figure 5C**). These metabolites were involved in 3 pathways: purine metabolism, beta-alanine metabolism and histidine metabolism. (**Figure 5D**) We further investigated the alternation of these significantly discriminative metabolites in both groups. (**Figure 5E**) The heat map demonstrated the averaged values of pre-CIRT and post-CIRT samples indicating that the significantly discriminative metabolites between two groups were all down-regulated after CIRT in the SIB group, but four out of eleven metabolites were up-regulated after CIRT in the non-SIB group. It should be noted that all 3 metabolites in purine metabolism were down-regulated to a greater extent in the SIB group than the non-SIB group.

## Discussion

Metabolic reprogramming is vital for the survival and growth of cancer cells. Plentiful metabolites have been found to have diagnostic, predictive, prognostic, or therapeutic values (36). A few former studies explored metabolomic changes after several kinds of therapeutic strategies. Uridine and six acylcarnitines showed a lower level after radical prostatectomy, while the level of other 16 metabolites, such as succinic acid and choline increased eight weeks after surgery (25). Tissue samples from high‐risk localized PCa patients who received neoadjuvant docetaxel and ADT represented down-regulation of many endogenous metabolic pathways and a general decline of metabolites (24). Metabolic pathways of nitrogen, pyrimidine, alanine, aspartate and glutamate and porphyrin were significantly altered after photon RT (26). While, the level of urine metabolites showed a dynamic increase and decrease during and after photon RT in PCa patients. Some bile acids and acylcarnitines were down-regulated, while 7,12-dioxolithocholic acid was significantly up-regulated. The metabolic changes after carbon ion and X-ray irradiation were not exactly the same. A larger number of urine metabolites, especially in amino acid metabolism, were significantly altered after CIRT in our study compared with photon pelvic RT (27). Each strategy had a unique effect on the metabolite profiles of PCa patients.

In this study, to profile metabolic changes in PCa patients who received CIRT and ADT and explore the metabolic effect of SIB CIRT by comparing two different CIRT strategies, we performed a metabolomic study on 23 PCa patients and compared metabolic effects induced uniform-dose-distribution CIRT and SIB CIRT.

The altered metabolite profiles of post-CIRT urine samples from PCa patients represent a unique cluster distinct from the pre-CIRT urine samples. CIRT with concurrent ADT showed a strong ability to suppress the metabolism in PCa. About 92.0% of metabolites significantly altered metabolites were down-regulated in both groups.

Alanine, aspartate and glutamate metabolism was the most significantly altered pathway after CIRT and ADT. One interesting thing is that PSMA, widely known for its application in functional imaging as a surface antigen, is also a member of N-acetyl-L-aspartyl-L-glutamate peptidase I (NAALADase) family, which involves in regulating the alanine, aspartate and glutamate metabolism pathway by cleaving the terminal poly glutamates connected with N-Acetyl-L-aspartate. The direct substrates of PSMA, such as N-acetylaspartylglutamylglutamate and N-acetyl-L-aspartate were significantly down-regulated or showed a trend to be down-regulated. Other substrates in this pathway like L-aspartate, L-asparagine, N-(L-arginino)succinate, and beta-citryl-L-glutamate were also significantly down-regulated. There were no significant differences in other metabolic pathways according to the criteria of FDR<0.05 and impact>0.2. Both groups showed a down-regulation of metabolites in this PSMA participated metabolic pathway, which indicated both schemes could nicely inhibit the metabolism of PSMA positive cells. It is worth noting that most metabolites in this pathway showed a trend to be suppressed to a lower level in the SIB group, which possibly meant that the SIB CIRT to the PSMA positive focal lesion may be more effective to inhibit the metabolism of PCa cells. This pathway was reported to be up-regulated in PCa tissues (37), and its dysregulation was highly correlated with the more malignant state (38, 39) and metastatic potentials in many malignancies (40–42). Meanwhile, PSMA is considered to be important for PCa carcinogenesis, for higher PSMA uptake on PET/CT is associated with higher PSA, GS, and d’Amico risk score, which often imply adverse clinical outcomes (43). The inhibition of PSMA and its metabolic pathway could be vital for better clinical outcomes, and this result points out the necessity of applying the technique of PSMA-PET/CT-guided SIB to CIRT.

Several clinical trials have worked on the efficacy and safety of the SIB technique using photon irradiation. Results of FLAME randomized phase III Trial (44) indicated that conventional fraction (77 Gy in 35 fractions) with a SIB (boost up to 95 Gy) to mpMRI visible intraprostatic lesion could improve biochemical control without increasing the risk of late GU and GI toxicity. 5-year biochemical disease-free survival was 92% in the SIB arm and 85% in the stander arm without SIB. The ^18^F-choline-PET/CT-directed SIB group had a trend to improved clinical results than the controlled group. The 5-year biochemical tumor control was 92% vs. 85% in the SIB vs. conventional groups (p=0.17). The 5-year OS was 100% and 88%, respectively (p=0.06) (45). Meanwhile, Schild et al. (46). compared the clinical outcome of patients treated with molecular imaging directed (^111^In Capromab pendetide (ProstaScint) scan) SIB with patients who did not receive SIB. The 5- and 10-year biochemical control rates for patients who received the SIB were 94% and 85%, respectively, while for patients who did not receive SIB were 86% and 61% (p=0.02).

All these clinical results concluded that the SIB technique was more effective and pretty necessary for localized PCa, yet studies seldom looked into the underlying mechanism. Up till now, the functional imaging-guided SIB technique has not yet been applied to CIRT according to our knowledge, and there have been few studies dug into the underlying mechanism. This is the first study using metabolomics to explore the distinction of the two CIRT strategies: SIB CIRT and the uniform-dose-distribution CIRT from the perspective of metabolism. Back to the results in this study, patients who received the SIB CIRT technique showed a greater extent of reduction after CIRT. The metabolite profiles clearly and intuitively showed that the SIB CIRT technique was more powerful to inhibit most metabolism of PCa than uniform-dose-distribution CIRT, which may be an explanation for better outcome of the SIB group in previous clinical trials (44–46).

As we further tried to explore the significantly discriminative metabolites between the two groups to find out the unique metabolic function of the SIB technique, eleven identified metabolites were found. It should be noted that there were three metabolites in purine metabolism pathways significantly discriminative between the two groups, and all of them showed a greater down-regulation in the SIB group compared with the non-SIB group. Purine, basic components of nucleotides, play an important part in DNA replication, RNA synthesis, and cellular bioenergetics (47). Abnormally high levels of purine can promote cell survival and proliferation. Inhibition of purine metabolism is critical to kill cancer cells (48, 49). Metabolomic study of urine samples from PCa patients and healthy volunteers indicated that purine metabolism was involved with carcinogenesis of PCa (50). Some metabolic products of purine were considered as biomarkers of radiation response. Nalbantoglu et al. (26) performed metabolomic analysis on PCa patients who received RT and found out that purine metabolism was significantly altered in serum samples after RT. Purine metabolism is also related to irradiation dose-effect and radiation sensitivity (51). It was significantly altered in the radioresistant cells after photons irradiation, while this pattern was not observed in the radiosensitive cell line. Purine metabolites were increased in RT-resistant cell lines when DNA damage occurred (52) after photon irradiation and the inhibition of *de novo* purine synthesis impairs DNA reparation (53) after irradiation. Alternation of purine metabolism could also be observed after carbon ion irradiation (54). In our research, several metabolites in purine metabolism were significantly discriminative between the two groups. As purine metabolism is highly related to DNA damage and repair, the inhibition of this pathway in the SIB group may cause the effect on impairing the DNA repair in cancer cells, especially RT-resistant ones. While, in our study, this pathway was significantly better inhibited in the SIB group than in the non-SIB group. This indicated that the SIB CIRT technique is necessary to inhibit purine metabolism, and it may be more effective than uniform-dose-distribution CIRT, making the SIB CIRT technique a promising and necessary strategy to cure localized PCa.

We also use ADT in our treatment, and ADT doesn’t cause exactly the same metabolic changes as radiation therapy. Previous studies (55, 56) on plasma and serum metabolomics after ADT suggest that metabolic changes induced by ADT mainly include steroid metabolism, ketogenesis, fatty acid metabolism, microbiome metabolism, lipid beta‐ and omega‐oxidation, and bile acid metabolism. However, our study is a urine metabolomic study, so there are certain differences with blood metabolism, and the metabolite changes are mainly concentrated in amino acid metabolism. The previous study (56) indicated, at six months after ADT, about 2/3 of metabolites were decreased and about 1/3 of differential metabolites were increased, while about 92% of metabolites in our study were down-regulated, so the combination of the two treatments is a better choice.

Still, this study is limited by its small sample capacity. The larger amount and long-term results need to be further investigated. As we used semiquantitative untargeted metabolomics as a preliminary exploration in this study, quantitative targeted metabolomics still needs to be further explored for its potential value in clinical practice.

## Conclusion

Metabolic dysfunction was one of the typical characteristics of PCa. The combination CIRT and the SIB technique showed a strong ability to suppress the abnormal metabolism in PCa. Most metabolites declined to extremely lower levels in the SIB group compared to untreated patients and even the non-SIB group. Both strategies could nicely inhibit the PCa associated PSMA metabolic pathway named alanine, aspartate and glutamate metabolism pathway, and the SIB group had even better performance. The SIB technique also showed its unique superiority to inhibit purine metabolism pathways. The SIB CIRT technique may be an effective and promising strategy for localized PCa.

## Data Availability Statement

The original contributions presented in the study are included in the article/**Supplementary Material**. Further inquiries can be directed to the corresponding authors.

## Ethics Statement

The studies involving human participants were reviewed and approved by Institutional Review Boards of Shanghai Proton and Heavy Ion Center. The patients/participants provided their written informed consent to participate in this study.

## Author Contributions

YP, QZ, and RN finished study design. QZ and YP finished experimental studies. YP and YS finished data analysis. QZ, PL, ZH, YP, WH, ZZ, and YD collected and proceeded patients’ samples and clinical information. YS, QZ, and YP finished manuscript editing. QZ, XG, and YS supervised the study. All authors read and approved the final manuscript.

## Funding

This article was supported by the Science and Technology Development Fund of Shanghai Pudong New Area (PKJ2020-Y52), the Natural Science Foundation of Shanghai (21ZR1481800).

## Conflict of Interest

The authors declare that the research was conducted in the absence of any commercial or financial relationships that could be construed as a potential conflict of interest.

## Publisher’s Note

All claims expressed in this article are solely those of the authors and do not necessarily represent those of their affiliated organizations, or those of the publisher, the editors and the reviewers. Any product that may be evaluated in this article, or claim that may be made by its manufacturer, is not guaranteed or endorsed by the publisher.

## Abbreviations

PCa, Prostate cancer; CIRT, Carbon ion radiotherapy; SIB, Simultaneous integrated boost; ADT, Androgen deprivation therapy; PPI, Permanent prostate implantation; UPLC-MS/MS, High-performance liquid chromatography, coupled to tandem mass spectrometry; CTV, Clinical target volume; GTV, Gross target volume; OS, Overall survival; LEM, local effect model; mpMRI, multiparametric magnetic resonance imaging; QC, Quality control; MetDNA, Metabolite identification and Dysregulated Network Analysis (http://metdna.zhulab.cn/); FC, Fold change; FDR, False discovery rates; PCA, Principal component analysis; PLS-DA, Partial least-squares discriminant analysis; GCPII, Glutamate carboxypeptidase II.
