# Supplementary material for: Carbon Ion Radiotherapy Induce Metabolic Inhibition After Functional Imaging-Guided Simultaneous Integrated Boost for Prostate Cancer
Source: Front Oncol. 2022 Jul 22;12:845583. doi: 10.3389/fonc.2022.845583 (PMC9354483; doi:10.3389/fonc.2022.845583)
Supplement: Supplementary file 1 [file DataSheet_1.docx]

**Supplementary Material**

**
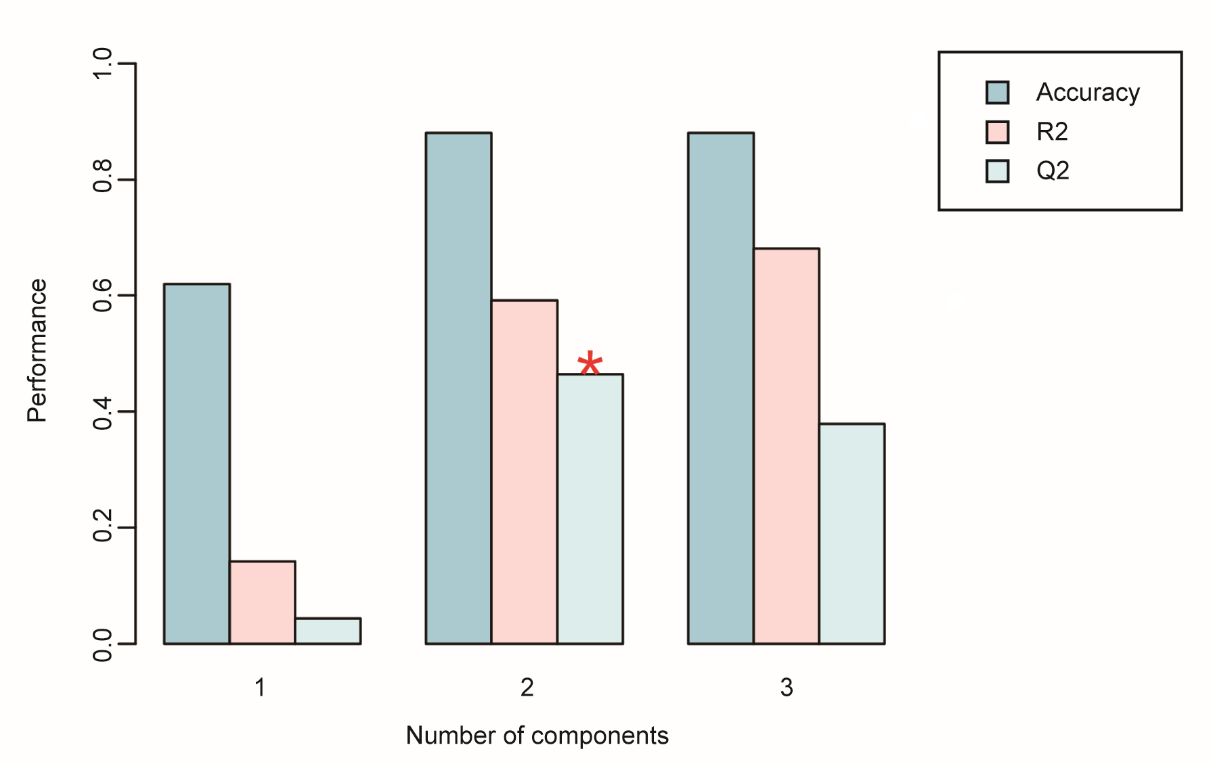
**

**Supplementary Figure 1.** PLS-DA cross validation details.


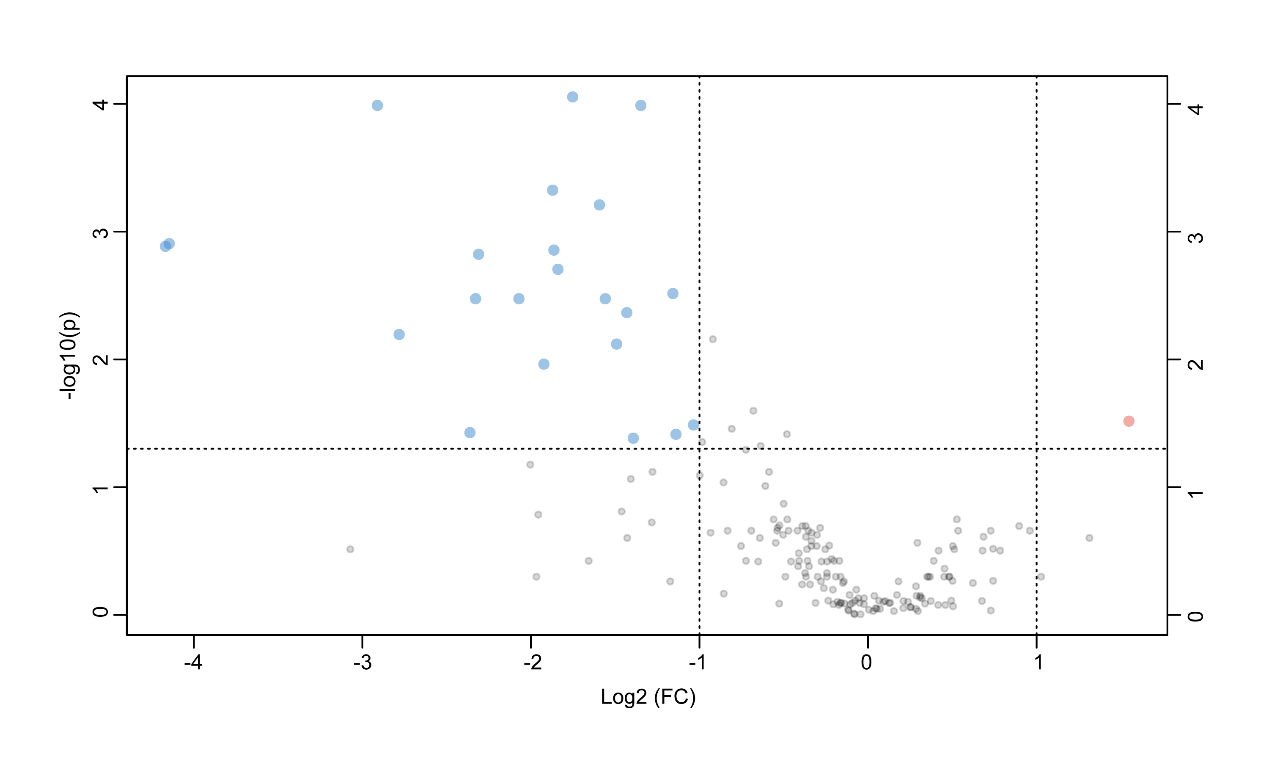


**Supplementary Figure 2.** The volcano plot of pre-CIRT and post-CIRT samples from all PCa patients in both groups. Blue and red dots represent significantly down-regulated and up-regulated identified metabolites, respectively (FDR<0.05, FC>2).

**Supplementary Table 1.** PLS-DA cross validation details.

| Measure | 1 components | 2 components | 3 components |
| --- | --- | --- | --- |
| Accuracy | 0.62 | 0.88 | 0.88 |
| R2 | 0.14109 | 0.59182 | 0.68101 |
| Q2 | 0.043201 | 0.46437 | 0.3791 |

**Supplementary Table 2.** Significantly altered metabolites after CIRT by the volcano analysis in all patients.

| Compound name | FC | log2(FC) | p.ajusted | -Log10(p) |
| --- | --- | --- | --- | --- |
| N-Acetylaspartylglutamylglutamate | 0.29681 | -1.7524 | 8.82E-05 | 4.0545 |
| D-Lysopine | 0.13296 | -2.911 | 0.000103 | 3.9875 |
| (2S,3R)-3-Hydroxybutane-1,2,3-tricarboxylate | 0.39288 | -1.3478 | 0.000103 | 3.9875 |
| L-Aspartate | 0.27331 | -1.8714 | 0.000474 | 3.3241 |
| 3-Hydroxy-N6,N6,N6-trimethyl-L-lysine | 0.33134 | -1.5936 | 0.000618 | 3.2094 |
| beta-Citryl-L-glutamate | 0.056478 | -4.1462 | 0.001242 | 2.9058 |
| Cystine | 0.055607 | -4.1686 | 0.001304 | 2.8846 |
| L-2-Amino-6-oxoheptanedioate | 0.27478 | -1.8637 | 0.001395 | 2.8555 |
| S-(Hercyn-2-yl)-L-cysteine S-oxide | 0.20157 | -2.3107 | 0.001505 | 2.8226 |
| D-Galactonate | 0.27935 | -1.8399 | 0.001972 | 2.7052 |
| 4-Carboxy-4-hydroxy-2-oxoadipate | 0.44825 | -1.1576 | 0.003051 | 2.5155 |
| 3-Indoleacetonitrile | 0.19908 | -2.3286 | 0.003349 | 2.4751 |
| N-(L-Arginino)succinate | 0.23797 | -2.0712 | 0.003349 | 2.4751 |
| N4-(Acetyl-beta-D-glucosaminyl)asparagine | 0.33941 | -1.5589 | 0.003349 | 2.4751 |
| 2-Aminomuconate | 0.37082 | -1.4312 | 0.004308 | 2.3657 |
| Cys-Gly | 0.14541 | -2.7819 | 0.006379 | 2.1952 |
| Trypanothione | 0.35555 | -1.4919 | 0.007582 | 2.1202 |
| N6-(L-1,3-Dicarboxypropyl)-L-lysine | 0.26372 | -1.9229 | 0.01089 | 1.963 |
| L-Histidinal | 2.9242 | 1.548 | 0.030415 | 1.5169 |
| D-Ribonate | 0.48786 | -1.0355 | 0.032577 | 1.4871 |
| N8-Acetylspermidine | 0.19452 | -2.362 | 0.037396 | 1.4272 |
| N(pi)-Methyl-L-histidine | 0.45395 | -1.1394 | 0.038526 | 1.4142 |
| S-Glutathionyl-L-cysteine | 0.38084 | -1.3927 | 0.041335 | 1.3837 |

**Supplementary information:**

In this research, ADT induce change of prostate volume was about 22%.
